# Whole heart DTI using asymmetric bipolar diffusion gradients

**DOI:** 10.1186/1532-429X-17-S1-P15

**Published:** 2015-02-03

**Authors:** Martijn Froeling, Gustav J Strijkers, Aart J Nederveen, Peter R Luijten

**Affiliations:** 1 Radiology, UMC Utrecht, Eindhoven, Netherlands; 2 Biomedical Engineering and Physics, Academic Medical Center, Amsterdam, Netherlands; 3 Radiology, Academic Medical Center, Amsterdam, Netherlands

## Background

Cardiac diffusion weighted imaging (DWI) using a spin echo sequence is challenging because of its high sensitivity to bulk motion. When the displacement or acceleration are constant and coherent but not equal for all spins within a voxel, it will cause signal attenuation due intra voxel de-phasing as an effect of first or second order moment encoding errors. This signal attenuation cannot be distinguished from that originating from diffusion weighting. To compensate for these effects bipolar gradients have been proposed that compensate for first order moment encoding [1]. However, this method will only work under the assumption of a uniform non-accelerated motion. Therefore, the aim of this study was to develop SE-based cardiac diffusion MRI protocol with second order moment nulling, thus also compensating for acceleration, and to compare its performance to that of Stejskal-Tanner and bipolar gradients waveforms.

## Methods

**Figure 1:**
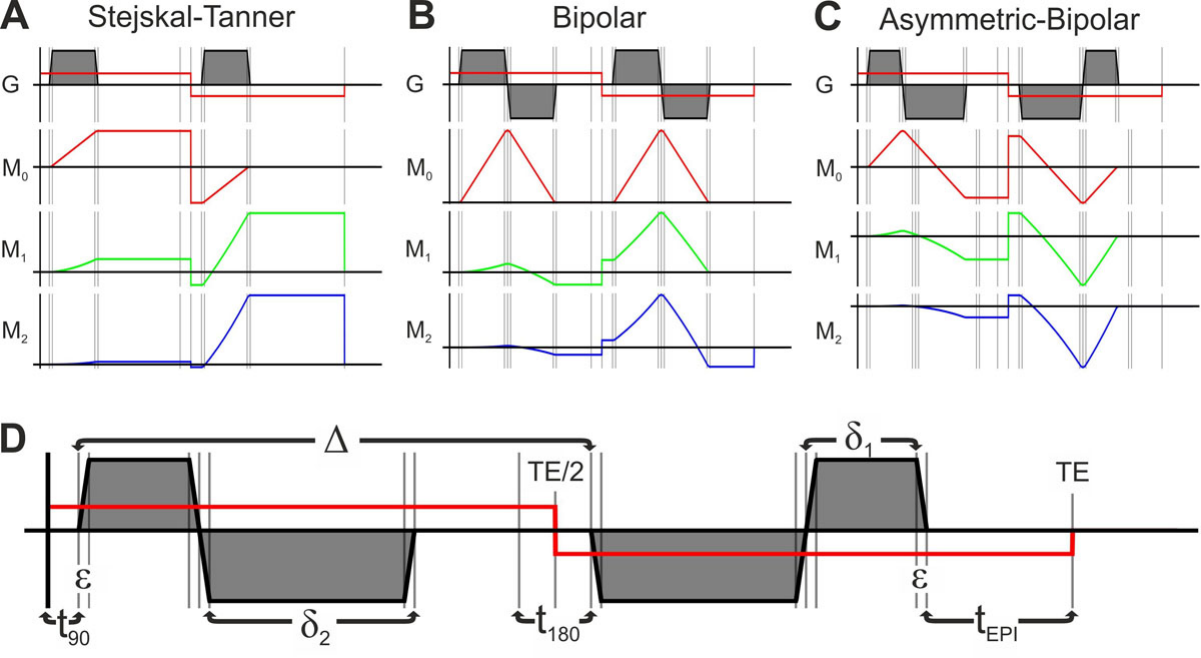
Diffusion weighed SE sequence with Stejskal-Tanner (A), Bipolar (B) and asymmetric bipolar (C) diffusion weighted gradients together with their gradient moments M_n_(t) (Red: M_0_; Green: M_1_; Blue: M_2_). D) The timing of the asymmetric Bipolar gradient waveform. Its first and second order gradients moments are zero when δ_1_ = (-Δ δ_2_ +δ_2_ ε)/(Δ + 2δ_2_ + ε).

Images were acquired with cardiac triggering and free breathing on a 3T scanner (Philips, Achieva) using a 16-channel coil (Torso XL). DWI was performed using a SE sequence in free breathing with Stejskal-Tanner, bipolar and asymmetric bipolar gradients (figure 1) and additional flow compensation [2]. The echo times were, 42, 60 and 66 ms, respectively. Further imaging parameters were; TR = 8 heart beats; FOV = 280 x 150 mm^2^ (using outer volume suppression); slices = 12 (2 interleaved 6 slice packages); voxel size = 6 x 2.5 x 2.5 mm^3^; acquisition matrix = 112 x 58; SENSE factor = 2; partial Fourier = 0.85; EPI train = 37; EPI duration = 23.2 ms; EPI bandwidth = 36 Hz/pix; averages = 4; trigger delay = 200 ms; gradient directions = 12; b-value = 400 s/mm^2^; Gmax = 62 mT/m; max slope = 100 mT/m/ms and acquisition time = 10 min.

## Results

**Figure 2:**
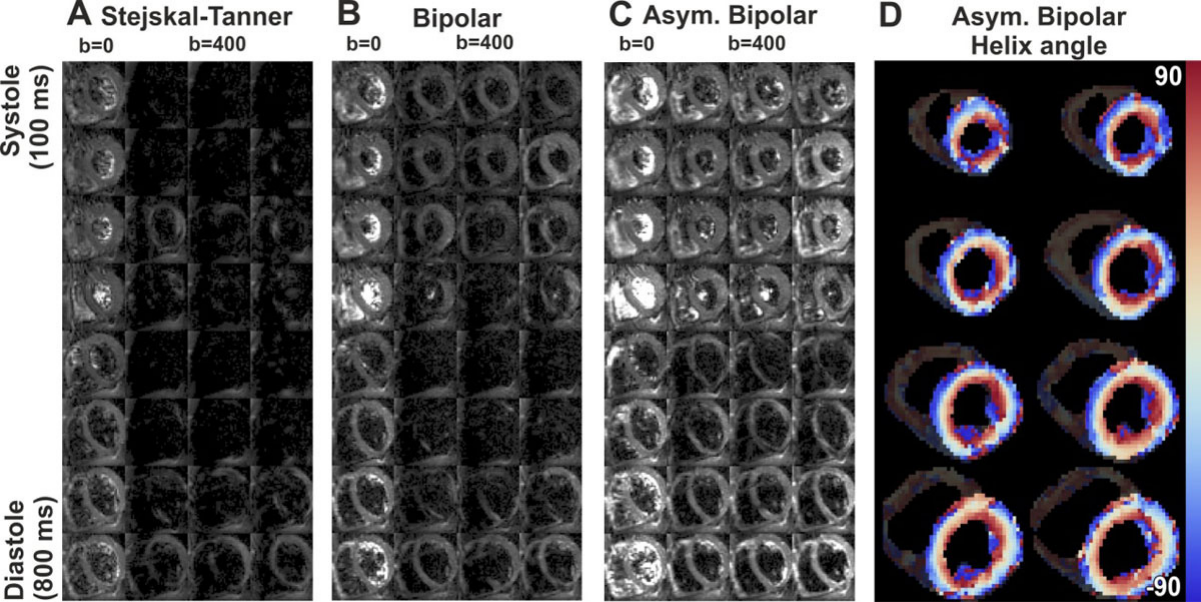
A-C) Diffusion weighted imaging using Stejskal-Tanner (A), bipolar (B) and asymmetric bipolar (C) diffusion encoding gradients in different cardiac phases (100 to 800 ms in steps of 100 ms). For each sub-figure the left column shows the un-weighted images (b = 0s/mm^2^) and the right three columns show the diffusion weighed images (b = 400 s/mm^2^) in the x, y and z directions, respectively. D) Helix angle maps calculated form DTI data acquired with asymmetric bipolar diffusion gradients.

DWI data acquired in different cardiac phases (100 to 800 ms) using Stejskal-Tanner, bipolar and asymmetric bipolar diffusion encoding gradients are shown in figure 2A-C. Signal dropout due to motion was reduced most with the asymmetric bipolar gradients (figure 2C). Maps showing the transmural changes in helix angle, which were calculated from data acquired with these gradients, are shown in figure 2D.

## Conclusions

In this study whole heart DTI using second order moment nulling diffusion gradients was performed. Using this approach we have shown that it is feasible to quantify the transmural helix angle for the entire heart. The DTI data was acquired in free breathing with a 10 min protocol, making this protocol well suited for clinical applications.

## Funding

N/A.
